# Impacting Binational Health through Leadership Development: A Program Evaluation of the Leaders across Borders Program, 2010–2014

**DOI:** 10.3389/fpubh.2017.00215

**Published:** 2017-08-21

**Authors:** Omar A. Contreras, Cecilia B. Rosales, Eduardo Gonzalez-Fagoaga, Celina I. Valencia, Maria Gudelia Rangel

**Affiliations:** ^1^Department of Community, Environment, and Policy, University of Arizona Mel and Enid Zuckerman College of Public Health, Tucson, AZ, United States; ^2^Division of Public Health Practice and Translational Research, University of Arizona Mel and Enid Zuckerman College of Public Health, Phoenix, AZ, United States; ^3^Mexico Section, U.S.-Mexico Border Health Commission, Tijuana, Mexico

**Keywords:** border health, binational health, leadership, health diplomacy, training

## Abstract

**Background:**

Workforce and leadership development is imperative for the advancement of public health along the U.S./Mexico border. The Leaders across borders (LaB) program aims to train the public health and health-care workforce of the border region. The LaB is a 6-month intensive leadership development program, which offers training in various areas of public health. Program curriculum topics include: leadership, border health epidemiology, health diplomacy, border public policies, and conflict resolution.

**Methods:**

This article describes the LaB program evaluation outcomes across four LaB cohort graduates between 2010 and 2014. LaB graduates received an invitation to participate *via* email in an online questionnaire. Eighty-five percent (*n* = 34) of evaluation participants indicated an improvement in the level of binationality since participating in the LaB program. Identified themes in the evaluation results included increased binational collaborations and partnerships across multidisciplinary organizations that work towards improving the health status of border communities. Approximately 93% (*n* = 37) of the LaB samples were interested in participating in future binational projects while 80% (*n* = 32) indicated interest in the proposal of other binational initiatives. Participants expressed feelings of gratitude from employers who supported their participation and successful completion of LaB.

**Discussion:**

Programs such as LaB are important in providing professional development and education to a health-care workforce along the U.S./Mexico border that is dedicated to positively impacting the health outcomes of vulnerable populations residing in this region.

## Introduction

The United States (U.S.)/Mexico border is a unique epidemiological unit (1). The La Paz Agreement defines the U.S./Mexico border region the 100 km both North and South of the international border (2). This geopolitical border definition for the U.S./Mexico is the most widely used designation in the U.S. (2). While the La Paz definition of the U.S./Mexico Border is important, it is also useful to consider the fluidity of the border experience under globalization (3), which problematizes traditional conceptualizations of binational border health. The 3,200 km U.S./Mexico corridor from California to the southern tip of Texas is comprised of 48 U.S. counties and 80 Mexican municipalities (4). The U.S./Mexico border is the busiest international land border of any border in the globe (3). Approximately, 11.5 million people reside along both sides of the border. This population is projected to double by the year 2025 (5). Communities along the U.S./Mexico border are economically and socially interdependent (3). The interdependence of these border communities has larger public health implications. The epidemiological, social, and geographic realities of the U.S./Mexico border region require a public health workforce trained to serve the demands of this community.

The Border Health Commission (BHC) was created under the George W. Bush administration to address border health challenges through the prioritization of the health indicators along of the U.S./Mexico border. *Healthy Border 2020* is a BHC initiative that focuses on public health issues specific to populations of the U.S./Mexico border region. Five strategic priorities of disease prevention and health promotion have been identified by the BHC as being of particular importance for tackling current disease burden trends and disparities across the U.S./Mexico border. These five strategic priorities are: 1. chronic and non-communicable diseases; 2. infectious diseases; 3. maternal and child health; 4. mental health and substance abuse; and 5. injury prevention (6).

Pan American Health Organization estimates suggest that approximately 15,000 cases of tuberculosis are reported yearly in the U.S./Mexico border (7). Tuberculosis rates in Arizona border counties have been found to be nine times greater than the general state rate (8). Although diabetes mortality has declined in the U.S., the national mortality rate in Mexico reached 65 and 47% in the border region from 2000 to 2010 (6). In 2015, the infant mortality rate in Mexico reached 11.3 per 1,000 live births, when compared to infant mortality rate of 5.6 per 1,000 live births in the United States (9). Despite the decline in overall suicide rate in the U.S./Mexico border region over the past decade, suicide remains relatively high in certain border states and communities (10) ranking as the 10th leading cause of death. Traffic accidents continue to be the leading cause of death for school-aged children in Mexico (10). In 2013, age-adjusted transport-related death rates ranged from 10.2 deaths per 100,000 in the U.S border states of California, 14.0 per 100,000 in Arizona, 17.3 per 100,000 in New Mexico, and 14.1 per 100,000 in Texas when compared to 12.3 per 100,000 nationally (11). These health indicators demonstrate the U.S./Mexico border as an anomalous epidemiological unit with public health priorities that differentiate the border region from its surrounding context. The public health work force of the border region must be uniquely equipped with a skill set specific to the needs of border populations.

## Leaders Across Borders (LaB) Program

The LaB program was created to build professional development among binational public health practitioners, health-care professionals, and paraprofessionals (12). The goal of LaB is to provide a comprehensive leadership curriculum to impart health diplomacy skills specific to binational contexts among its participants. Health diplomacy for the purposes of this project is defined as the ability to communicate and negotiate public health issues across other sectors including but not limited to: international affairs, non-governmental organizations (NGOs), law, and medicine to contribute to shaping health outcomes (13).

The LaB program is designed for professionals with at least 5 years of field experience in the U.S./Mexico border region. Selected individuals have demonstrated a commitment to upholding the promotion of health equity across the continuum of care. Twenty professionals (U.S. *n* = 10; Mexico *n* = 10) from the U.S. and Mexico border states are recruited and competitively selected through professional networks and affiliations, listservs, academic institutions, and through the U.S./Mexico BHC yearly. Individuals interested in participating in LaB must complete a formal application that consists of formal letters of support from immediate supervisors, personal statement outlining professional experience in border health initiatives, and an online application. A selection committee reviews all of the applicant’s materials. Applicants are evaluated and selected by the selection committee *via* consensus. LaB selects one cohort of participants annually.

The program curriculum covers a diverse set of topics identified as mechanisms for improving health-care processes in the binational region (12). A total of eight modules constitute the curriculum. These eight modules target action learning leadership, cross-border communication, individualized and interpersonal relations in forming strategic partnerships, managerial and organizational skills, personal and interpersonal effectiveness leadership, and international health (Table 1). The LaB curriculum and leadership development involves 160 h of in-person and distance learning facilitated by an individual identified by the LaB coordinating body (Table 1). Sixty hours of the program are completed in three separate in-person meetings. The remaining curriculum is completed through the group’s preferred video conferencing software (i.e., Skype, Google Hangouts, WebEx, etc.). LaB participants are assigned into groups based on individual interests included in their application materials. To provide the opportunity to apply the newly acquired skills, LaB participants engage in a didactic learning experience. The purpose of the didactic learning experience is to solidify the leadership curriculum by providing the LaB participants a group exercise where they practically apply the skills taught in the LaB curriculum (Table 1). Twenty hours of the LaB curriculum are devoted to group projects. Groups sign an agreement charter that outlines group expectations, project goals, and timelines for project deliverables. Groups are encouraged to meet (in-person or online) for additional hours as needed for the completion of projects. Assigned facilitators are required to attend all group meetings.

**Table 1:** Leaders across borders (LaB) curriculum.

**LaB curriculum and competencies** – Introduction to epidemiology in U.S./Mexico border region (10 h) – Health diplomacy (10 h) – Management and leadership for regional health policies and programs (40 h) – Analysis and improvement of health-care processes in the border region (30 h) – Personal leadership skills (50 h) – Binational action-learning team projects (20 h)

**Topics** – Enneagram-learning your own leadership style – Action learning and leadership – Public Health Law in Mexico and the United States (U.S.) – Binational collaboration—part 1 – Fostering collaboration and leadership – Binational collaboration—part 2 – Leadership—part 1: what is it and why it is important in binational work? – Health conditions and epidemiology in the northern border and Healthy Border 2020 – Global health diplomacy and policy development – Leadership and its role in health diplomacy – Leadership—part 2: how to lead and negotiate? – Development and process of effective leadership – Mexico and U.S. health-care systems – Advanced leadership-application of all LaB training materials – Power nets-overcoming obstacles and leveraging resources in binational work

*Curriculum, competencies, and topics presented through the extent of 160 h*.

## Methods

Leaders across borders accepted the first cohort of participants in 2010. A total of 80 individuals have participated in LaB from 2010 to 2014. LaB was not offered in 2012. A total of four LaB cohorts had completed the program by 2014 when this evaluation was conducted. In 2014, all LaB participants were contacted by email correspondence to participate in a program evaluation. Institutional Review Board deemed the project to be exempt from Human Subjects review.

A 25-item survey instrument (Appendix B in Supplementary Material) was designed and collected with University of Arizona Qualtrics software (2017 Qualtrics LLC, Provo, UT, USA). The participants were informed that all information would be collected anonymously and participation was voluntary. Email reminders for participants to complete the evaluation questionnaire were sent three times over the course of 2 months. All descriptive statistics, population demographics, and survey frequencies were performed on STATA 13.0 (College Station, TX, USA). No qualitative analyses were conducted for this study.

## Results

A total of 40 individuals contacted participated in this evaluation (*n* = 40). The countries of origin of the evaluation participants were: 15 participants from Mexico (*n* = 15) and 25 from the U.S. (*n* = 25). The geographic distribution of participants in the U.S. is: 32% (*n* = 8) of LaB participants were from California and Texas; 16% (*n* = 4) from Arizona; and 20% (*n* = 5) from New Mexico. The majority of participants from Mexico (60%, *n* = 9) were from the state of Baja California, Norte. The remaining geographic distribution of participants from Mexico is: 13.33% (*n* = 2) from Sonora and Chihuahua; 6.67% (*n* = 1) from Nuevo Leon and Tamaulipas. No respondents from the state of Coahuila were identified. There were 62.5% (*n* = 25) more female participants than males in this participant sample. The median age among the evaluation participants was 48.5 years old (Table 1).

Thirty-seven percent (*n* = 15) of participants held leadership positions in health governmental agencies at either the state or municipal level (Table 2). Other employers were public health and primary care organizations including non-profit organizations, community health centers, hospitals/health-care organizations, and academic educational institutions. Several participants held more than one employment position within various sectors of the community (Table 2). Sixty percent (*n* = 24) of the participants rated the utility of the LaB course materials as “very well” or “well,” while 62.5% (*n* = 25) of participants agreed that course activities enhanced their work and preparation for their field of practice (data not shown).

**Table 2:** Leaders across borders (LaB) participant demographics.

Characteristics	Number of participants (*N* = 40)
**Year of LaB participation^a^**	
2010	9
2011	10
2013	7
2014	14
**Gender**	
Female	25
Male	15
**Age (years)**	
Mean age	46.5
Median age	48.5
**Area of employment at time of LaB participation^b^**	
State or local health government	15
Association, foundation, voluntary, non-governmental organization, or other non-profit organization	11
Hospital or other health-care facility (non-community health center)	9
University or other academic institution	7
Community health center	5
Consulting firm	2
Pharmaceutical, biotech, or medical device firm	2
Other	2
**Leadership/management position**	
Yes	35
No	5

*^a^There was no LaB cohort in 2012*.

*^b^Participants were not limited to selecting only one option for current place of employment*.

Approximately 67.5% (*n* = 27) of the participants attributed LaB as the impetus for increasing their participation in binational projects along the U.S./Mexico border. The binational projects reported in the evaluation were: chronic and infectious disease prevention, enhancement of epidemiologic surveillance systems, binational health consortiums and councils (COBINAS de salud), and binational training for community health workers were among the collaborative projects identified by past LaB participants.

The professional skills acquired and refined from LaB participation were acknowledged by participants’ employers. When asked whether the successes of the LaB training were recognized by their employers, 57.5% (*n* = 23) of participants reported yes. Approximately 82.5% (*n* = 33) of the participants reported being provided sufficient time from work to successfully complete LaB.

The LaB core curriculum emphasizes the importance of binational partnerships and collaborations. Participants responded to a series of questions that measured their level of binational engagement. Binational engagement was defined by the evaluation as those who have experience in border health initiatives resulting from their participation in the LaB program (Table 3). Eighty five percent (*n* = 34) of the LaB sample believed their binational involvement increased since participating in the LaB program (Table 3). Binational involvement included: fostering border relationships, engagement of mutual projects, and having an understanding of people’s different cultures, languages, and personalities. To successfully continue building the pipeline of public health and health-care professionals in the border region, the LaB program also evaluated participants’ willingness to engage in future binational projects post-LaB participation (Table 3). Almost 93% (*n* = 37) of the LaB sample were interested in participating in future binational projects while 80% (*n* = 32) indicated interest in other binational initiatives. Additionally, 80% (*n* = 32) of LaB participants agreed to serve as mentors, while 70% (*n* = 27) were interested in serving as LaB facilitators and other staff (data not shown). Given the value of this training and leadership program, approximately 88% (*n* = 35) were interested in building a network of former LaB participants for the continuous sharing of information and collaboration in projects pertinent to the U.S./Mexico border.

**Table 3:** Leaders across borders (LaB) program evaluative measures.

Measures	Number of participants (%)
**Are you collaborating in binational projects?**	
Yes	27 (67.5)
No	13 (32.5)
**Was the material you were exposed to during LaB useful in your job?**	
Yes	39 (97.5)
No	1 (2.5)
**Do you attribute your skill set in binational collaboration and communication, health diplomacy, leadership, and sociocultural to participating in LaB?**	
Yes	36 (90)
No	4 (10)
**Were sufficient resources (time off work) made available to you by your organization to participate in LaB?**	
Yes	33 (82.5)
No	6 (15)
Unknown	1 (2.5)
**Are your successes recognized and shared by your organization since participating in LaB?**	
Yes	23 (57.5)
No	16 (40)
Unknown	1 (2.5)
**Do you believe your level of binationality has improved since participating in LaB?**	
Yes	34 (85)
No	4 (11)
Unknown	2 (5)
**Since participating in LaB, have you increased your number of contacts and enhanced network?**	
Yes	33 (82.5)
No	4 (10)
Unknown	3 (7.5)
**Would you be interested in the following?**	
**Mentoring LaB graduates**	
Yes	31 (77.5)
No	1 (2.5)
Possibly	8 (20)
**Proposed binational initiatives**	
Yes	32 (80)
No	3 (7.5)
Possibly	5 (12.5)
**Participate in future binational projects**	
Yes	37 (92.5)
No	1 (2.5)
Possibly	2 (5)
**Engaging a network of past LaB participants**	
Yes	35 (87.5)
No	2 (5)
Possibly	3 (7.5)
**Promote LaB in other institutions**	
Yes	32 (80)
No	1 (2.5)
Possibly	7 (17.5)

## Limitations

Only 50 percent (50%; *N* = 40) of LaB participants opted to participate in the program evaluation. The response rate could be emblematic of a potentially biased sample of participants. This analysis accounted for four cohorts from 2010 to 2014 (excluding 2012). Future studies need to be conducted to determine the extent of the LaB program from 2010 to 2016 and its impact on binational and border health collaborations. A robust sample population will help facilitate a thorough analysis of over 100 participants in the LaB program to date. No formal qualitative analyses were conducted for the scope of this manuscript. Also, this study did not draw any comparisons between LaB participants and other health professionals in the U.S./Mexico border, who did not participate in this leadership program.

## Discussion

As indicated by the evaluation findings, LaB is an initiative that is successful in increasing the integration of a binational paradigm among the public health work force program participants. LaB program provides training to the health professional workforce along the U.S./Mexico border. It requires a key group of public health and health-care leaders to understand the needs of its communities. One study of 163 participants of the public health workforce determined that specialized trainings along the U.S./Mexico border are vital in assuring the quality and accessibility of health services (14). Furthermore, specialized trainings have shown to positively impact public health programming on both sides of the border (15). The U.S./Mexico border, as paralleled in other binational contexts globally, demonstrates the importance of leadership development and practice to understand the contextual factors of cultural, economic, geographic, and political across various sectors in society (16).

Public health curricula should be designed to enhance leadership competencies and facilitate performance in public health practice (17), specifically in the border region that faces unique public health challenges as evident in the epidemiological surveillance data. Leadership and professional development in public health and other areas of health services requires reinforcement of corresponding competencies with continued education (17). The LaB’s curriculum is centered on a set of themes that tackle health priorities of the U.S./Mexico border. Programs that promote collaborative and leadership attributes have the ability to engage health-care professionals in interdisciplinary projects to improve public health while building alliances, partnerships, and coalitions to positively impact the health of populations being served (18). Other leadership programs, for example in Europe, have tailored leadership modules to address chronic diseases (19). The European Credit Transfer System and its course modules emulate the LaB curriculum and its integration of public health and leadership content delivered *via* as face-to-face and on-line (19).

Cross-national public health leadership models are limited or have not been established. The U.S./Mexico border demands a workforce that is trained across various themes and disciplines. Effective public health leaders and practitioners require skills that affect constructive changes in health-care systems and work across numerous disciplines to achieve progress in solving complex public health problems (19). The U.S./Mexico border faces ongoing public health challenges. Thereby, the creation of the BHC and its continued support for programs such as LaB are imperative in undertaking public health problems and training a competent workforce.

Participants indicated that, overall, they found their participation in LaB to be rewarding. Participants noted LaB’s focus on needs of border populations was especially relevant to their leadership development as members of the border health field. Participants indicated that training that focused on border communities, mobility, and needs of this geographic region set LaB apart from other leadership development programs.

Participants of the LaB program indicated an increased knowledge of binational work and increased networking for the sustainability of ongoing binational projects that align with *Healthy Border 2020* priorities. Additionally, all participants were granted permission from employers and time off regular work duties to partake in a 6-month program that resulted in enhanced binational collaborations and formed partnerships with academic institutions and other border community based-organizations. The BHC strives to support opportunities that build a stronger workforce of binational leaders along the U.S./Mexico border, and the LaB program achieves that. The LaB should be expanded by the BHC as a permanent, binational investment in the future of both countries.

As the LaB curriculum evolves, the following topics are considered to be refined and integrated into the current curriculum to provide participants with other competencies pertinent to border health. Themes that include: evaluation, assessment, and program planning and implementation for binational driven projects; knowledge and availability of social and health services in both countries for vulnerable families; research theory; grant writing and development training; transborder communication and the integration of marketing and social media skills; engagement of policymakers at the federal, state, and local levels; population health; and advanced skills in epidemiology are considered vital to the professional development of LaB. Ultimately, building the skill set and leadership of health professionals can only positively impact the health outcomes and health status of border community residents.

The border region requires a workforce that is able to serve the demands of its community. Continued and professional development and training should be warranted among health systems and organizations operating in the border region. Leadership courses should be built into the practices of these organizations to sustain a workforce that understands the dynamics of the border community. Continued funding for leadership programs, such as the LaB should be a strategic priority embedded in future health border initiatives by the BHC to sustain the training, leadership, and professional development of practitioners and leaders serving U.S./Mexico border communities.

## Author Contributions

OC is a doctoral candidate in Public Health Policy and Management at the Mel and Enid Zuckerman College of Public Health and principle author of this manuscript. He also conducted the descriptive analysis in addition to crafting tables and figures. CR is the Associate Dean for the University of Arizona Mel and Enid Zuckerman College of Public Health, Phoenix Programs. CR’s vast experience in border health policy and former member of the United States Border Health Commission was valuable in drafting this manuscript. As second author, CR was pivotal in the data collection and dissemination of survey to study participants. Furthermore, CR served as editor for this manuscript and primary advisor to OC. EG-F and CV were critical in providing guidance on statistical and descriptive analyses. Their expertise in statistical analyses was sought for the development of this manuscript. MR is the Executive Director of the Mexico Section of the U.S./Mexico Border Health Commission. As senior author and expert in border health leadership and training, MR has spearheaded the development of the leaders across borders program. Her input was critical on every component of this manuscript.

## Conflict of Interest Statement

The authors do not have any conflict of interests to declare. This article was written in the absence of any commercial relationships that could be construed as a potential conflict of interest.
