# Supplementary figures and images for: Impacting Binational Health through Leadership Development: A Program Evaluation of the Leaders across Borders Program, 2010–2014
Source: Front Public Health. 2017 Aug 21;5:215. doi: 10.3389/fpubh.2017.00215 (PMC5566989; doi:10.3389/fpubh.2017.00215)

Appendix 1

**Evaluation Data Collection Instrument**


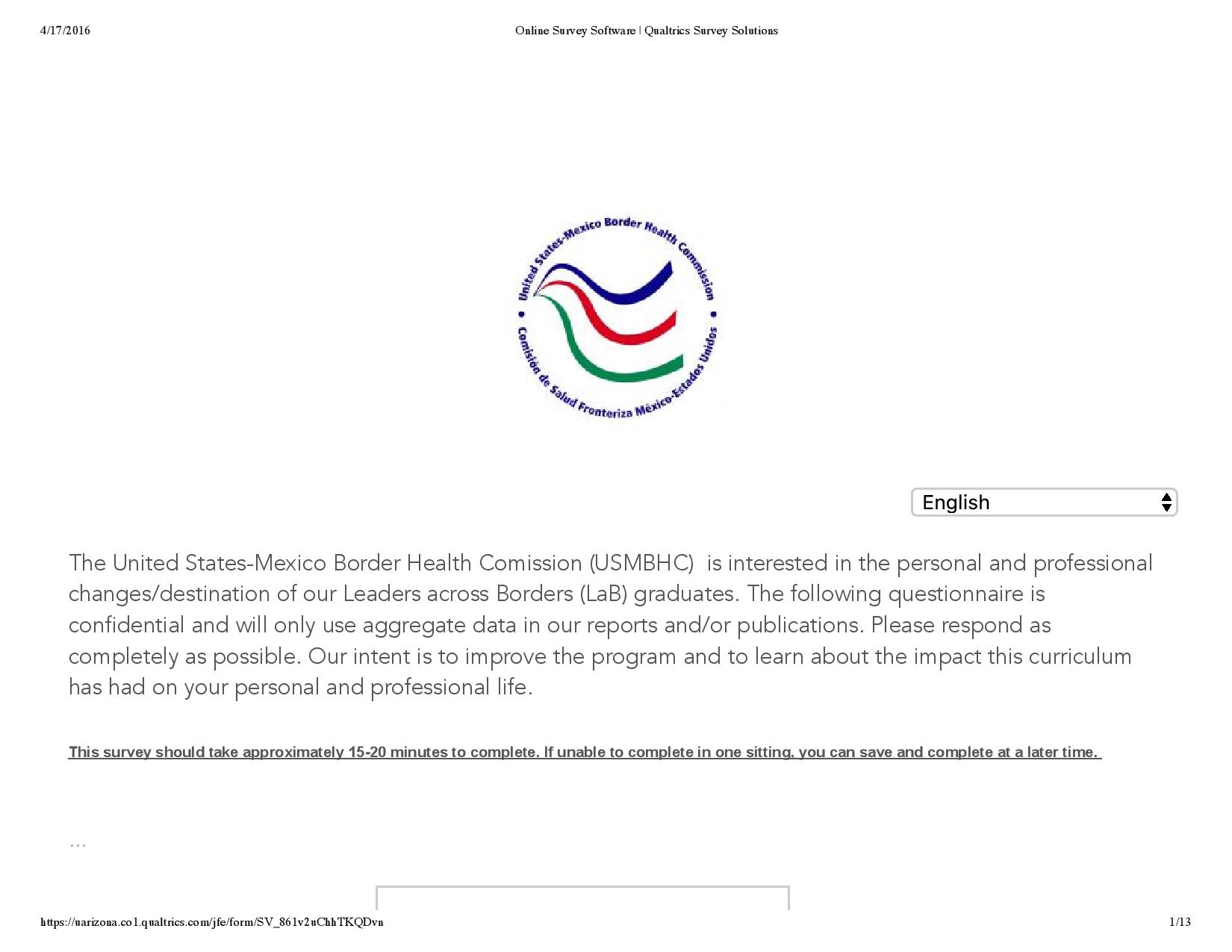


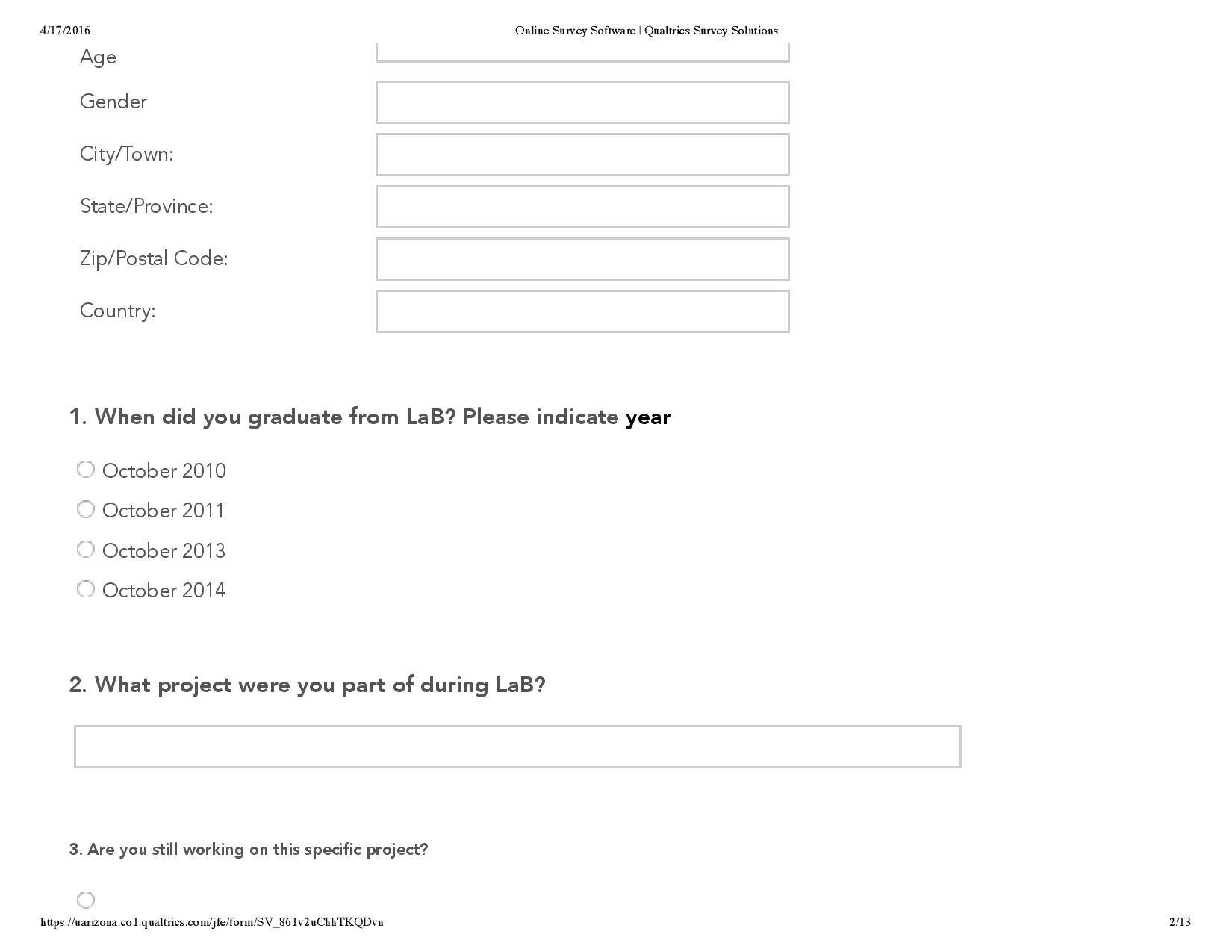


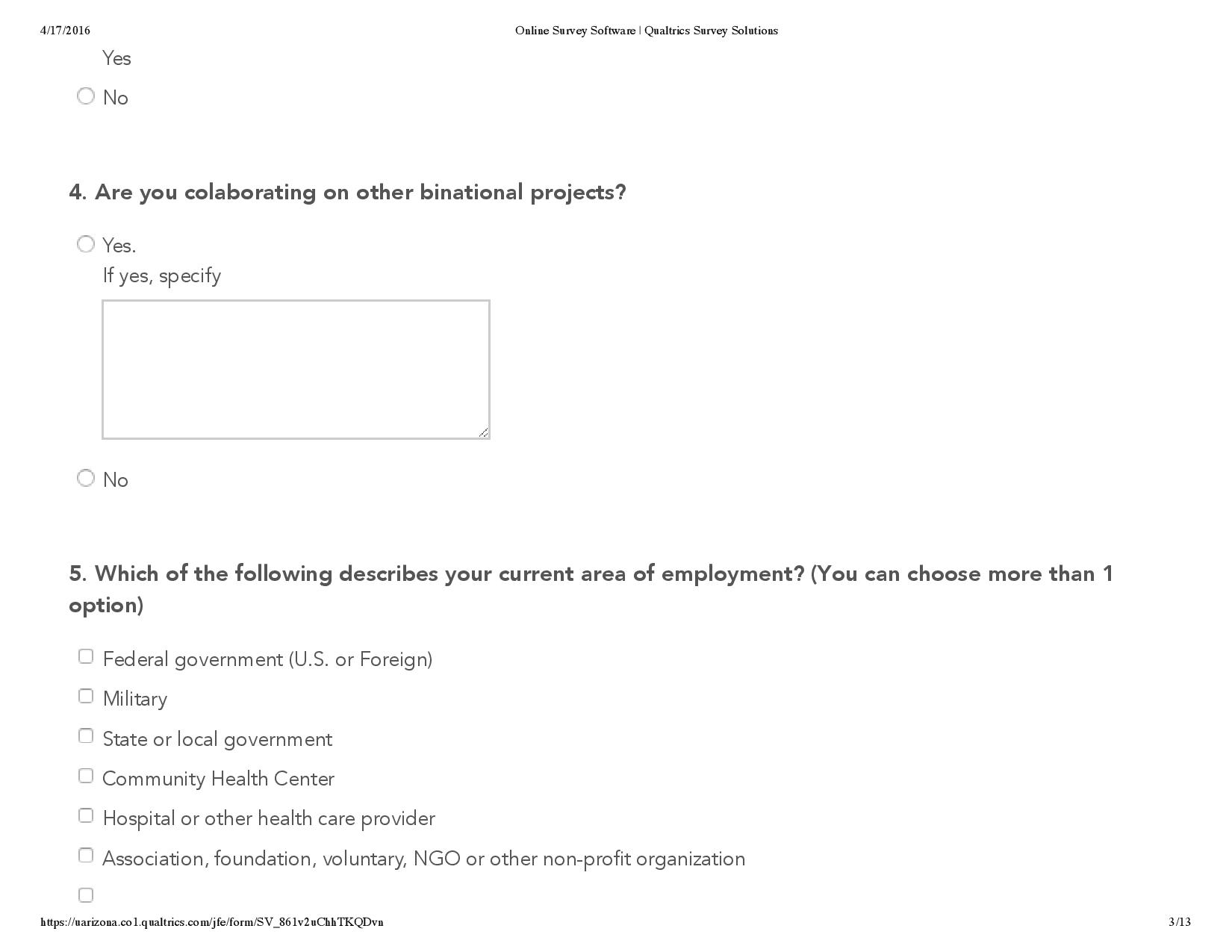


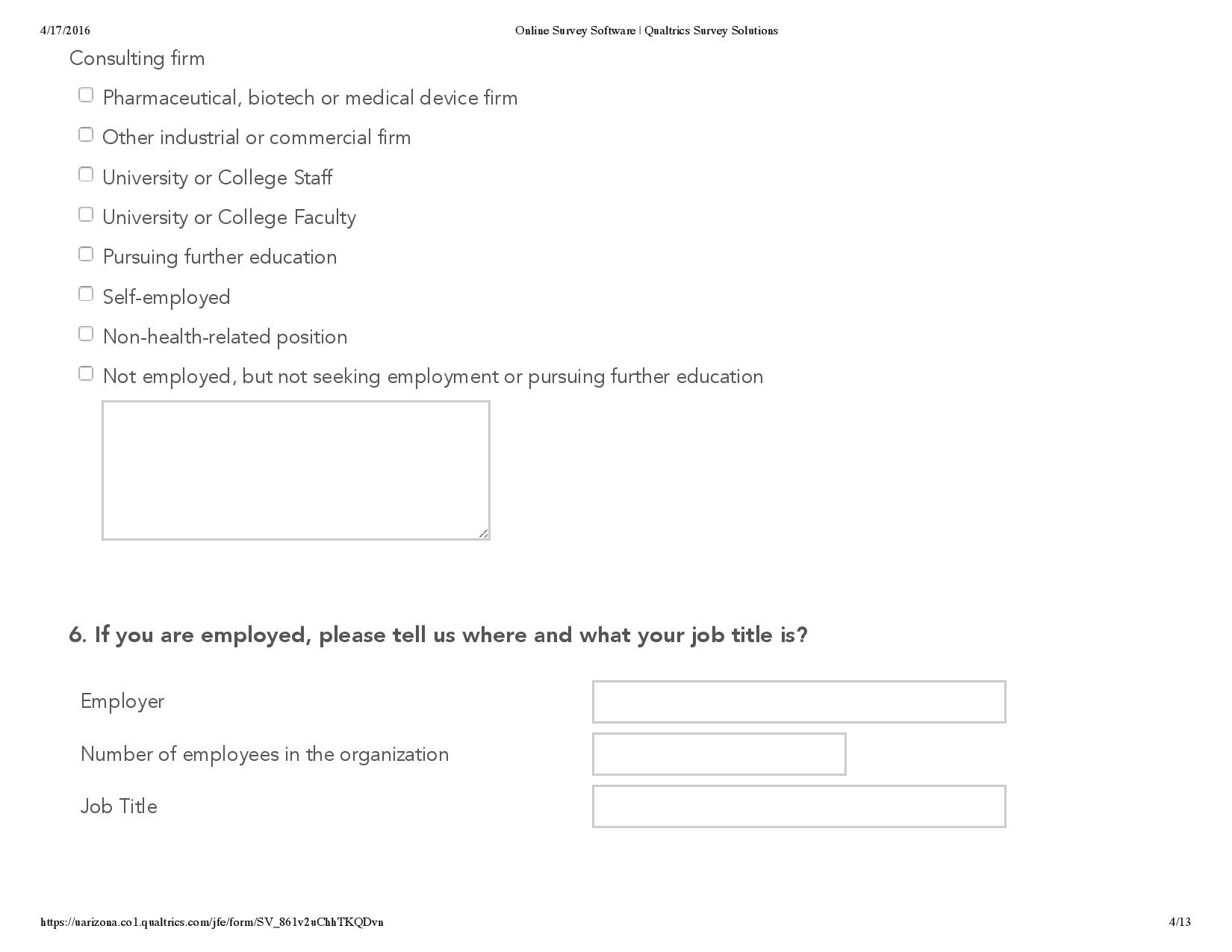


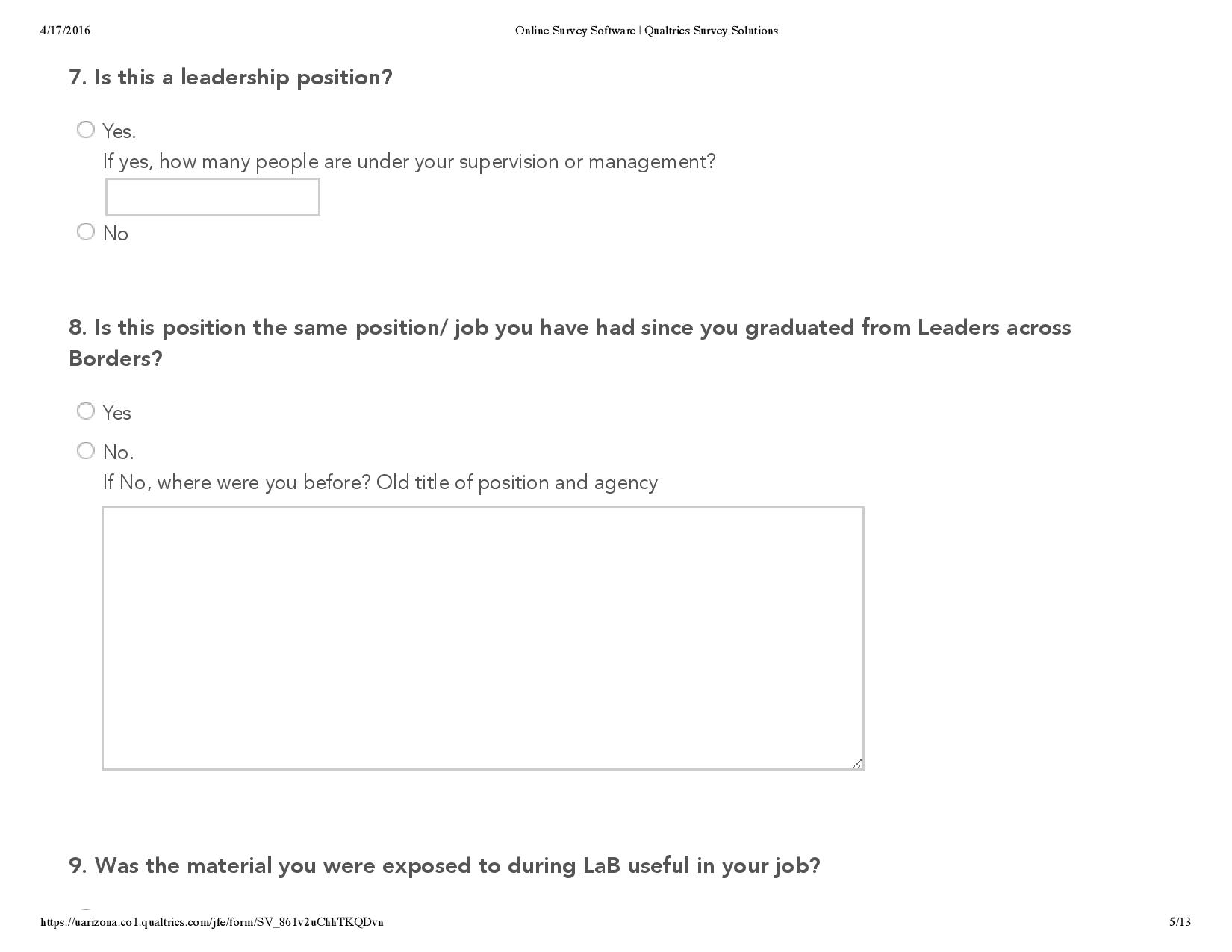


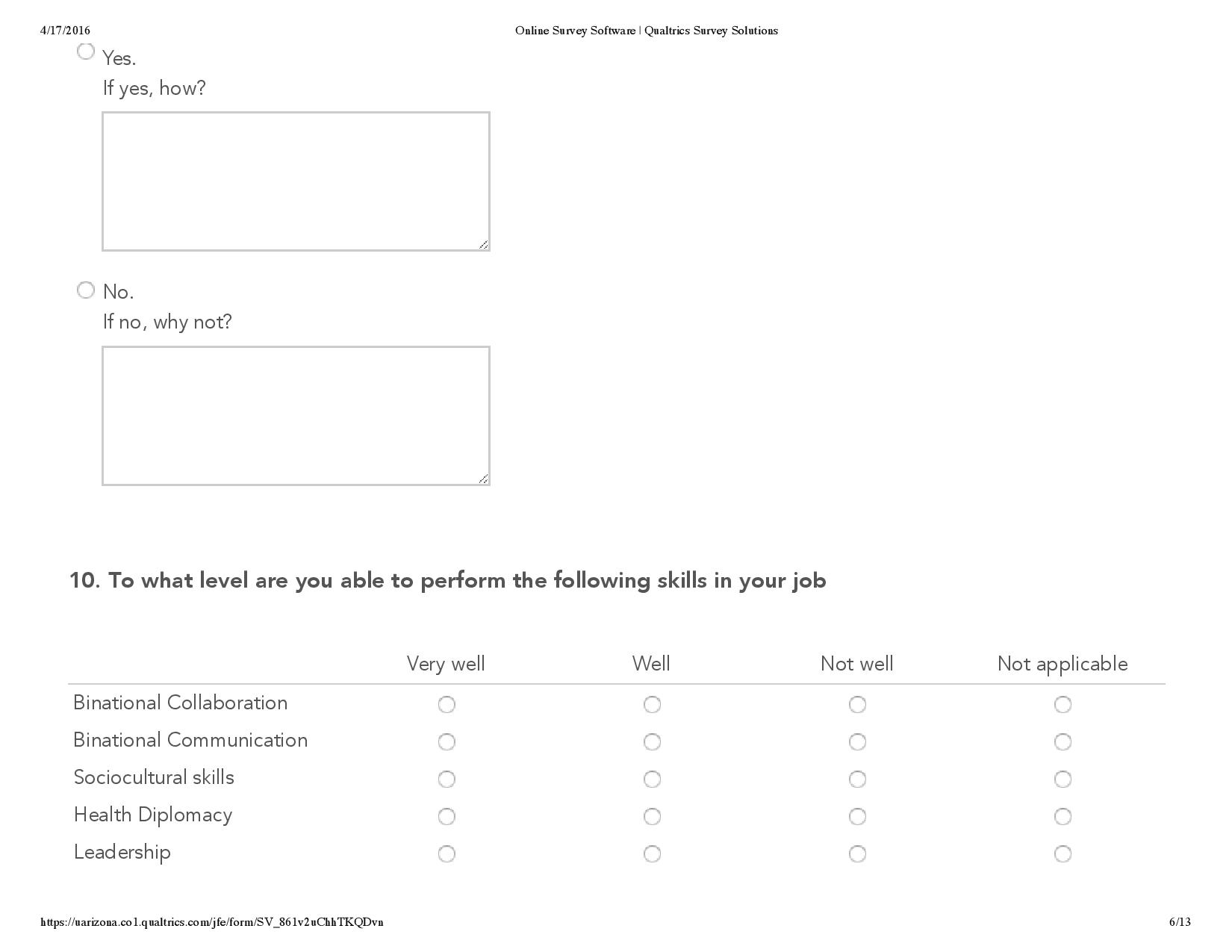


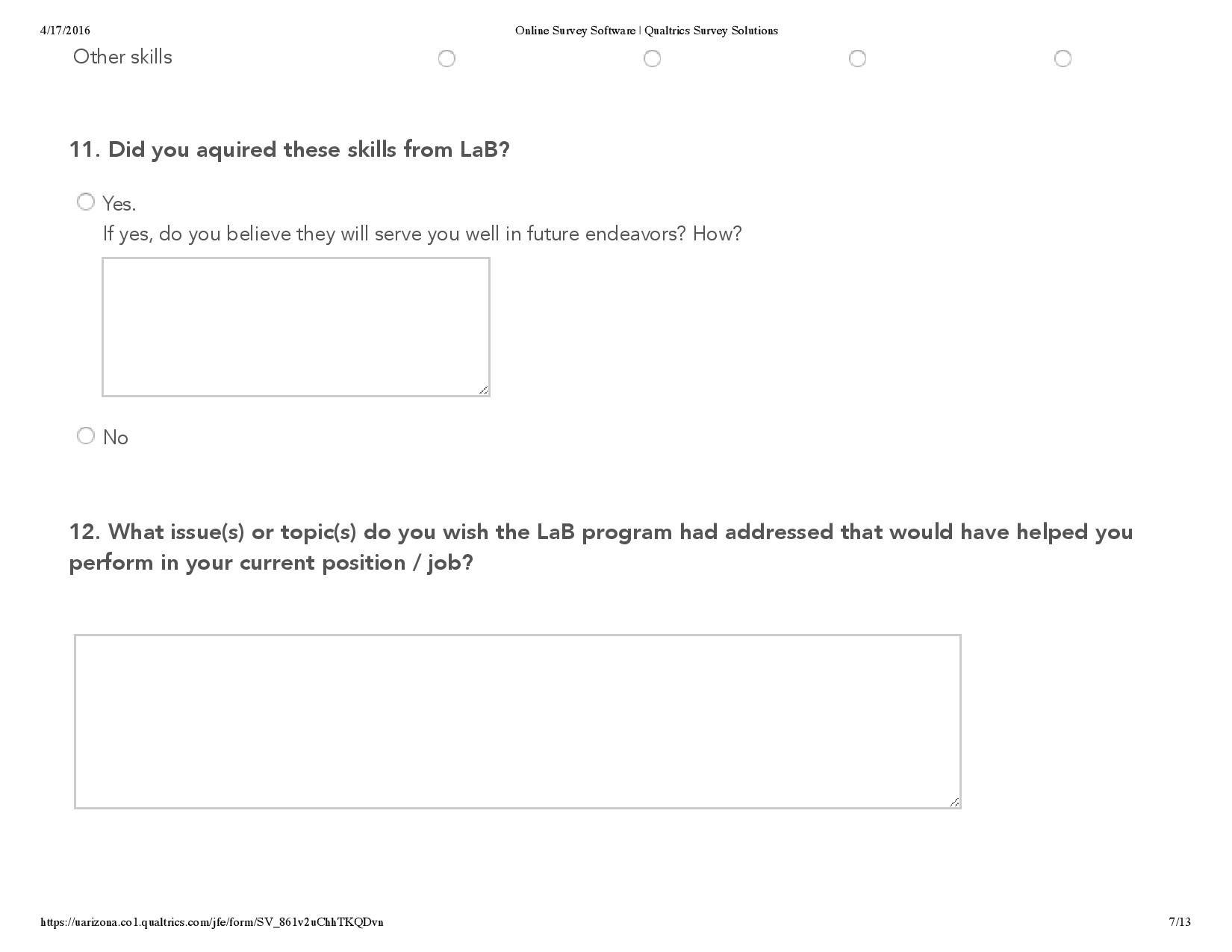


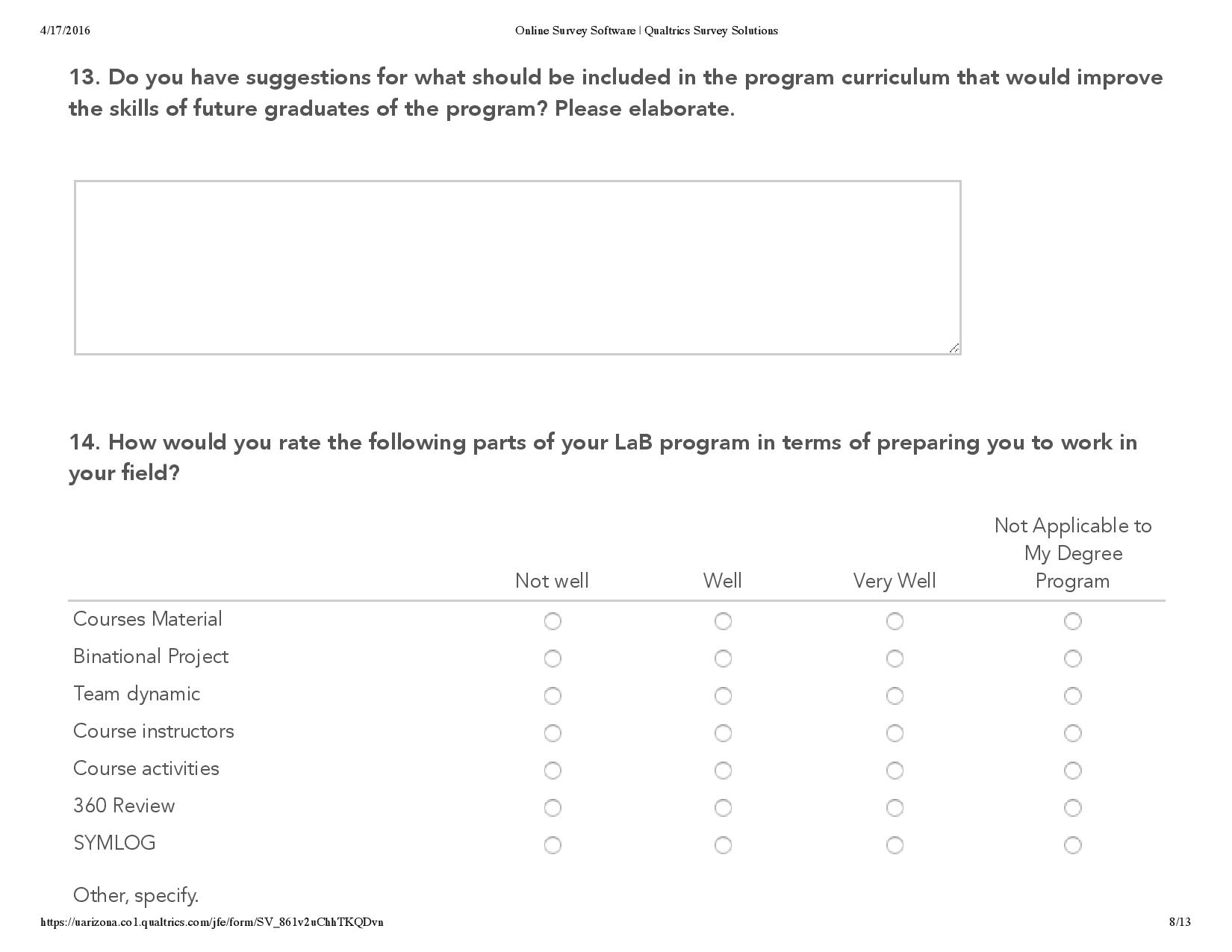


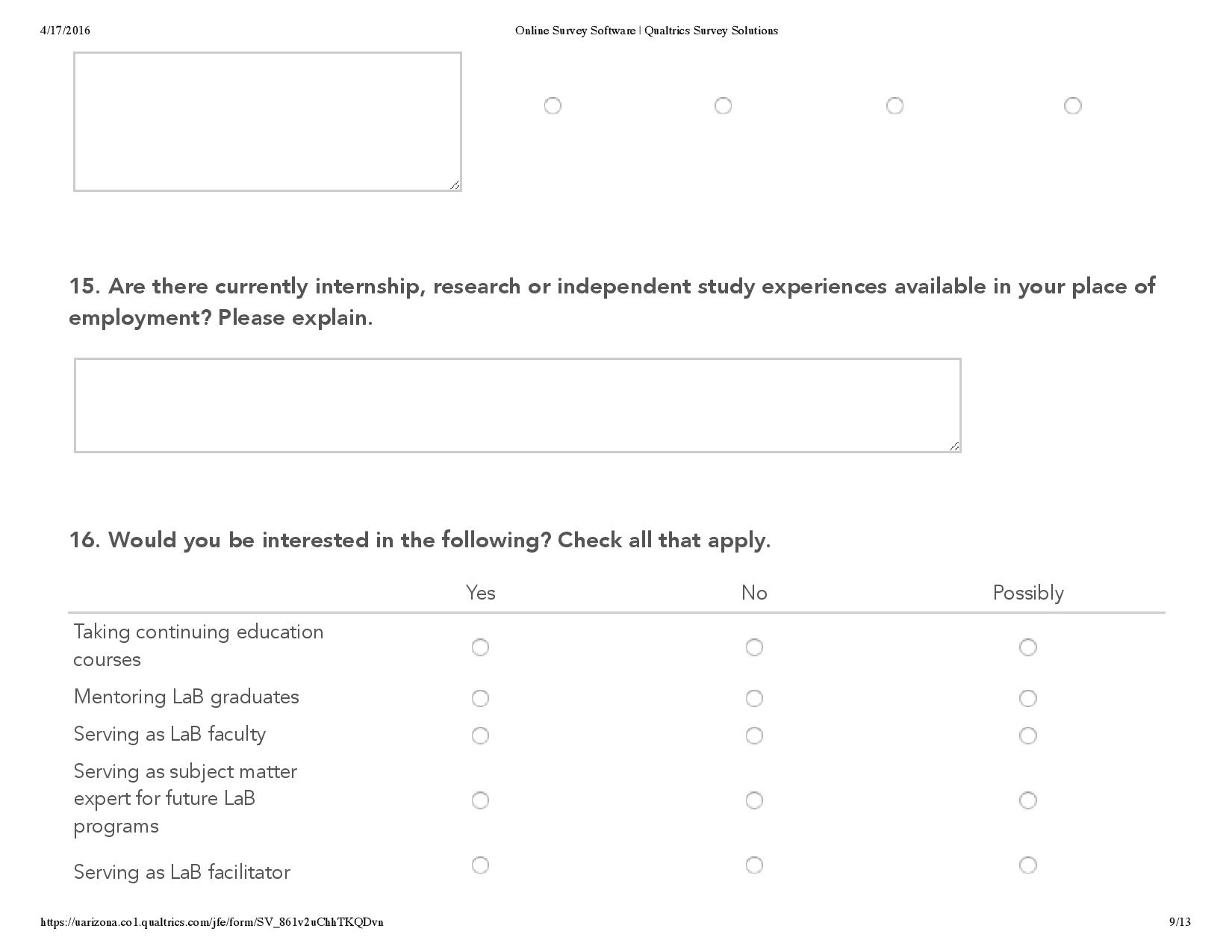


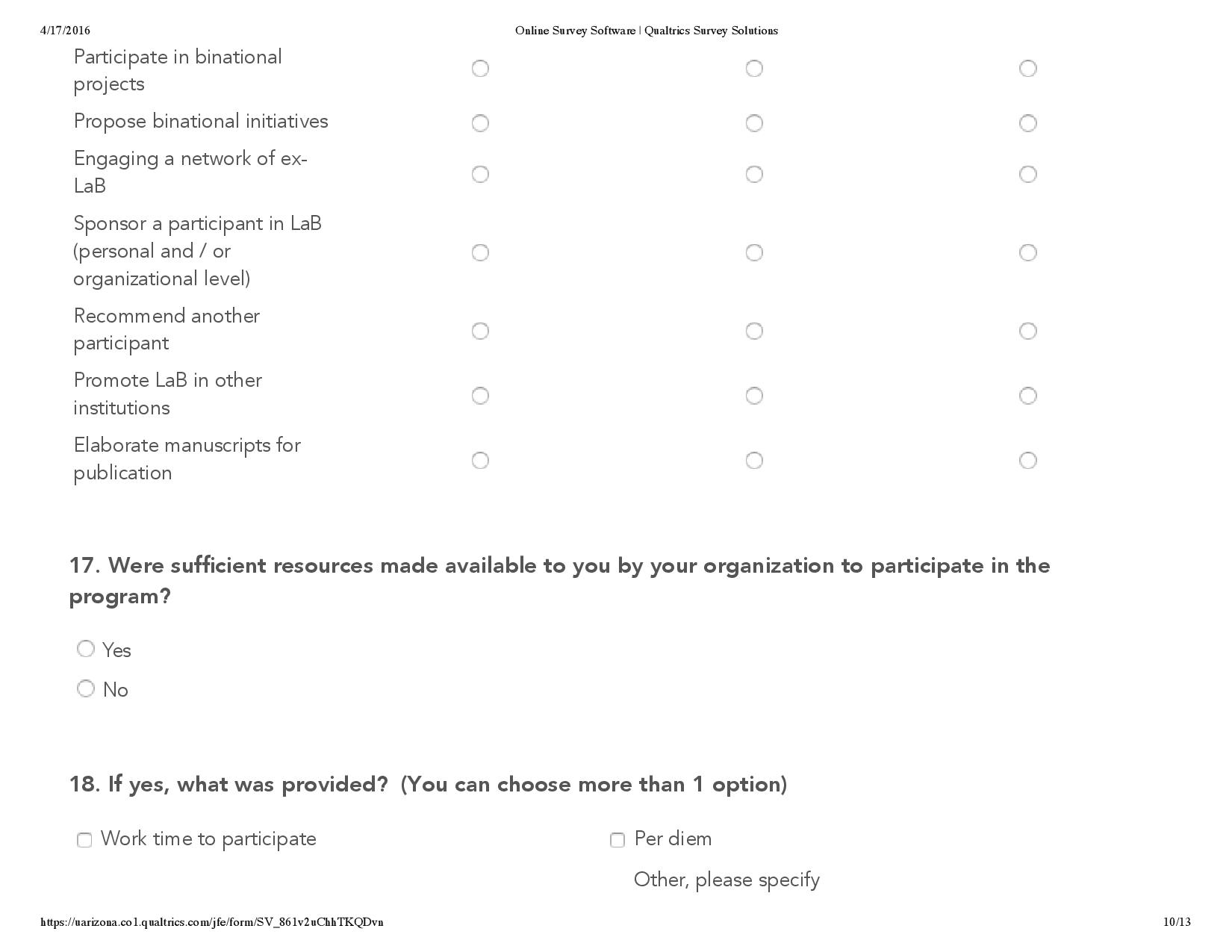


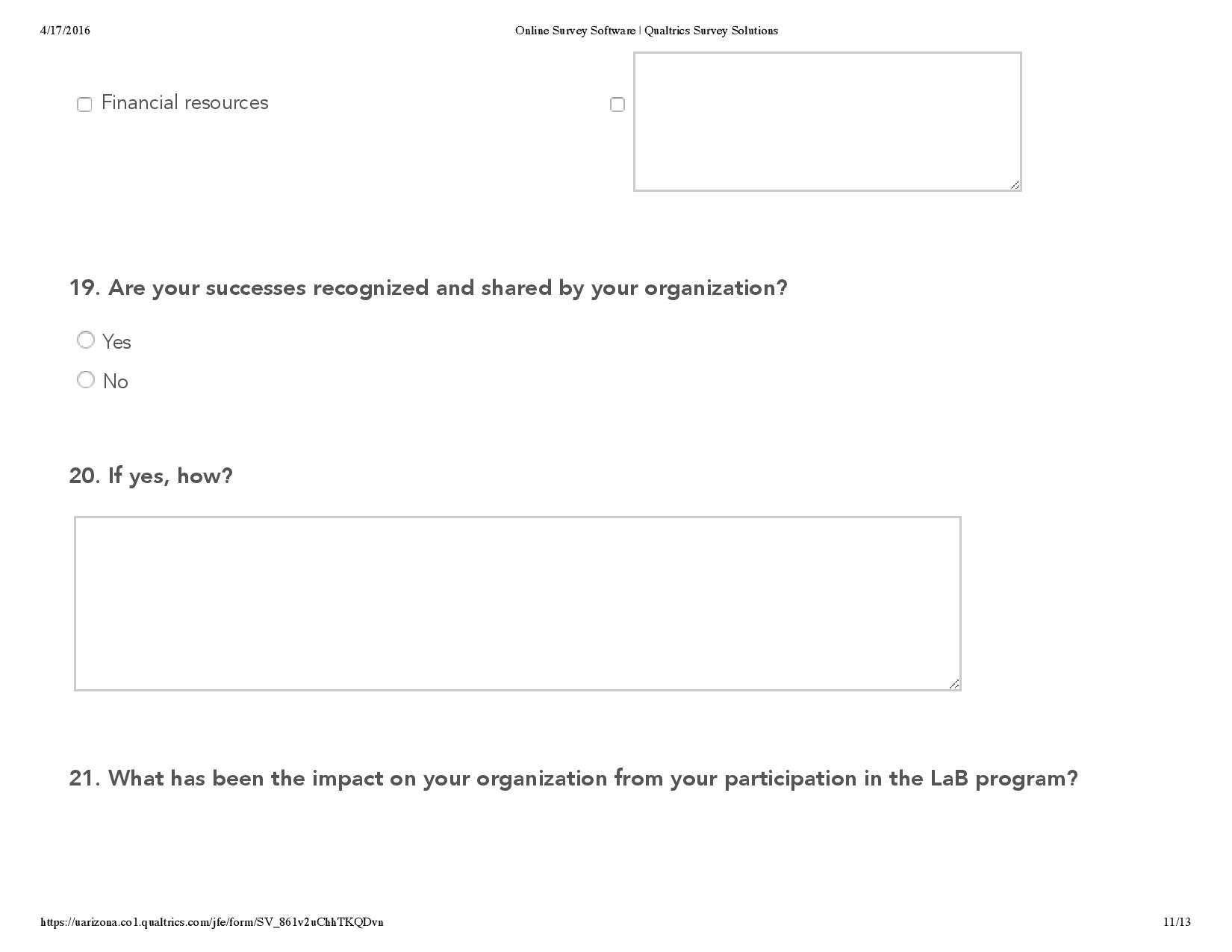


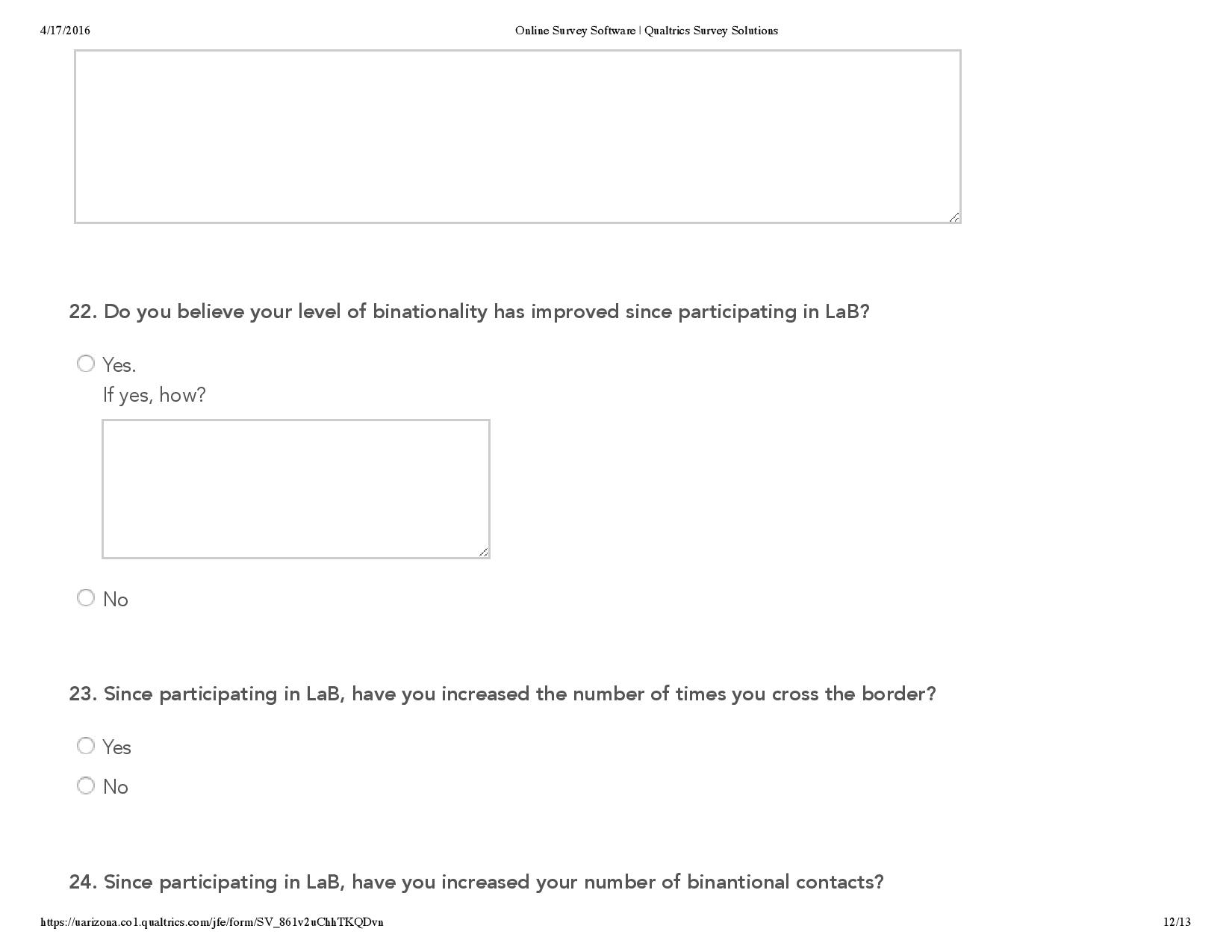


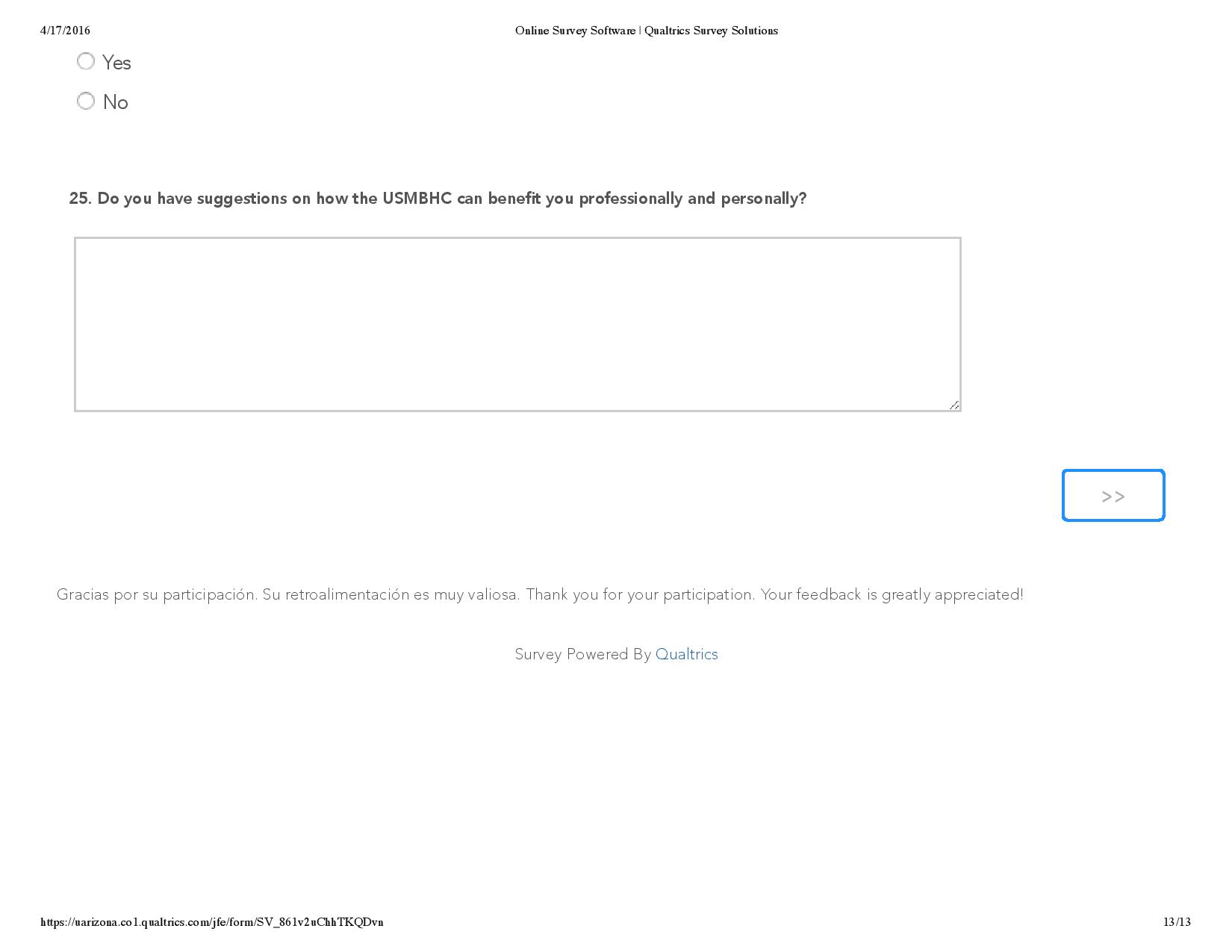

Supplement: Supplementary file 1 [file Data_Sheet_1.DOCX]
